# Interaction and thermal studies on graphene oxide in NC/DEGDN/GO nanocomposites

**DOI:** 10.1039/c9ra07717k

**Published:** 2019-10-31

**Authors:** Prima Kharisma Indra Yahya, Mohammed Moniruzzaman, Philip P. Gill

**Affiliations:** Centre for Defence Chemistry, Cranfield University, Defence Academy of the UK Shrivenham, Swindon SN6 8LA UK m.moniruzzaman@cranfield.ac.uk +44(0)1793785391

## Abstract

Before considering the uses of graphene oxide (GO) in nitrate ester-based materials for performance and safety improvement, its interaction, compatibility and dispersion with the host matrices need to be well understood. This work addresses the interaction and dispersity of GO with nitrocellulose (NC)/diethylene glycol dinitrate (DEGDN)-based nanocomposites. The GO and DEGDN were successfully synthesised and characterised. The NC/DEGDN proved to be a good hosting matrix for the dispersion of GO nanosheets. Analysis of atomic force microscopy (AFM) showed that the thicknesses of dispersed GO were in the range of 1–4 nm suggesting that the GO in the nanocomposite consists of 1–2 layers for a 0.5% w/w GO containing nanocomposite and 2–4 layers for a 0.75% w/w nanocomposite. ATR-FTIR spectroscopy analysis established red-shifting of 744 to 752 cm^−1^ for the O–NO_2_ bond stretching vibrations, indicating bond stabilization by donor electron from the GO. The Raman spectra analysis showed GO peaks blue-shifting and broadening which is attributed to hydrogen bonding interaction between GO sheets and –NO_2_ groups. The activation energy of nitrate ester decomposition of NC/DEGDN/GO nanocomposites increases as a function of GO content from 167 kJ mol^−1^ and reaches a maximum of 214 kJ mol^−1^ for a 0.5% w/w GO loading. This suggests an improvement of the nitrate ester bond stability. These findings open a new direction to the application of GO in nitrate ester-based materials for increased stability, safety and shelf life.

## Abstract 2

Before considering the use of graphene oxide (GO) in nitrate ester-based materials for improved safety and performance its interaction, compatibility, and dispersion with the host matrices needs to be well understood.

## Graphical abstract

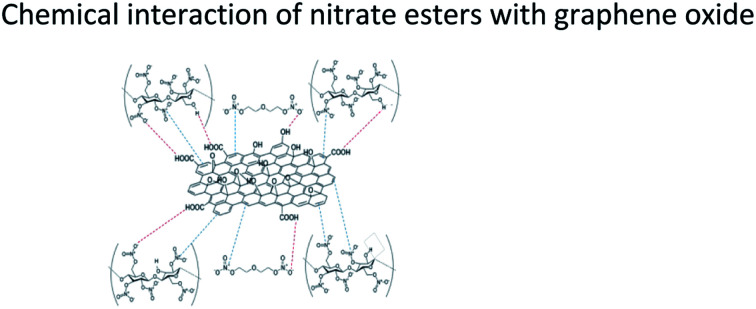

## Introduction

Graphene oxide (GO) is a hydrophilic oxygenated form of 2D lattice sp^2^-bonded carbon atoms. Since the invention of graphene, tremendous attention has been focused on research to establish its potential in the application of solar cells,^1,2^ biosensors,^3,4^ optical, electrical and thermal conductive applications. It is one of the strongest materials ever, with 1 TPa Young's modulus and 130 GPa ultimate strength. Graphene also has a very high surface area of 2630–2965 m^2^ g^−1^.^5^ The unique properties of graphene allow it to be a good reinforcement for polymers, composites,^6^ and to date graphene and its derivates have been applied in various polymers to achieve high reinforcement efficiency.^7–9^

Due to its high surface area, thermal and electrical conductivity and good optical transmittance graphene and GO have become the materials of interests in defence applications. The application of graphene and GO in the energetic fields is fairly new. A number of research articles have been published on improving the burning rate of propellants, desensitisation of primary explosives and secondary explosives.^10–15^ There are huge interests in exploring the possible applications of graphene and graphene derivatives to nitrate esters-based materials in order to improve their stability, safety and shelf life. Graphene and GO was observed to improve tensile strength, Young's modulus and elongation at break of NC,^13^ and also improve the laser ignition capability and burning rate of NC.^16^ Furthermore, the thermal decomposition and combustion behaviours of ammonium nitrate/GO propellant was observed to be improved. None of the published articles in the energetic fields reported the interactions of GO with the nitrate ester-base nanocomposites that triggers the aforementioned effects.

The unique structure and physical properties of graphene and its derivatives can be tailored with the various parameters of energetic materials by understanding several key factors such as degree of dispersion, orientation and interfacial adhesion.^8,17^ Due to the cohesive interaction of graphene and GO in water and organic solvents it is very difficult to disperse in organic materials, limiting their commercial application.^18^ In order to incorporate graphene into host matrices such as NC and other energetic materials, the compatibility and process chemistry must be understood. This can be achieved by producing homogeneously dispersed graphene and/or its derivatives within a matrix.^19^ To optimise the physical, thermal, mechanical, combustion and kinetic properties of NC based nanocomposites, the solubility of GO needs to be maximized in a common solvent. The solvent needs to either dissolve the energetic ingredients or one of the ingredients can act as a solvent as well as a component of the energetic nanocomposite formulations. While covalent interaction ensures a strong and irreversible bond between the polymer and GO, the weaker non-covalent approach avoids the introduction of sp^3^ defect sites on the GO surface. These non-covalent interactions of GO and polymer influence the physical, mechanical and electronic properties of GO/polymer nanocomposites. Although some properties such as burning rates, laser ignitability and stability of GO-NC compositions have been studied,^13,16^ to the best of our knowledge no published work addresses the interaction of GO with nitrate ester-based compositions. At low temperatures nitrate esters undergo a slow decomposition due to hydrolysis of the O–NO_2_ bond. This hydrolysis has a low activation energy (60–80 kJ mol^−1^) causing safety concerns. Therefore increasing the activation energy for the O–NO_2_ bond dissociation is of great importance to the lacquer and defence industries.

Graphene acts as electron donor and the addition of graphene or GO to the electron deficient nitrate ester groups is believed to increase the activation energy of O–NO_2_ bond improving the stability and safety.

The decomposition kinetics of nitrate ester-based formulations, are studied by thermally aging and calculating the Arrhenius parameters to assess possible changes in the activation energy. This data is required for energetic materials and their related compounds for safe life assessment.^20^

This article reports on the chemical interaction of GO with a NC/DEDGN matrix. It evaluates how GO affects the activation energy of nitrate ester decomposition in a double base propellant formulation. The dispersion behaviour of GO in NC/DEGDN composition was evaluated using atomic force microscopy. The interaction between GO and nitrate esters was established using FTIR and Raman spectroscopy and the findings are discussed. The activation energy of GO-modified and unmodified formulations is also reported.

## Experimental

### Materials and methods

All solvents and reagents were purchased from Sigma-Aldrich and used without further purification unless otherwise mentioned. Graphite materials was supplied by Cambridge Nanosystems.

### Graphene oxide synthesis

Graphene oxide synthesis was conducted by chemical oxidation of graphite following a modified Hummers' method.^21,22^ A typical synthesis method is: graphite flakes (1.0 g) were added to concentrated sulphuric acid (36 ml) and stirred for 1 h at room temperature. Fuming nitric acid (12 ml) was slowly added to the ice-cooled reaction mixture followed by continuous stirring. After cooling down (∼5 °C), potassium permanganate (5 g) was slowly added with stirring. The mixed slurry was stirred at room temperature for 12 h, followed by addition of hydrogen peroxide (30%, 6 ml) with production of a bright yellow solution with bubbles.

The solution was stirred for 2 h at room temperature and then allowed to settle for 24 h. The resultant yellow slurry was centrifuged and then washed, after which the supernatant liquid was washed in a solution consisting of deionized water (250 ml) with hydrochloric acid (37%, 1 ml) and hydrogen peroxide (30%, 10 ml). After stirring for 2 h at room temperature, the solution was filtered through a glass sinter and the remaining solid washed with of deionized water (250 ml) and hydrochloric acid (37%, 1 ml) and hydrogen peroxide (30%, 10 ml). This process was repeated three times. Afterwards, the yellow slurry was further washed with deionized water until the pH reached neutral. The collected product was dissolved in deionized water at a concentration of 0.5 g ml^−1^ and sonicated for exfoliation for 3 h. Graphene oxide was dried under vacuum prior to use.

### Diethyleneglycoldinitrate synthesis

Synthesis of diethyleneglycol dinitrate was conducted following a previously reported method.^23^ Nitric acid (99.5%, 4 ml) was added dropwise to sulphuric acid (98%, 4 ml) in a 50 ml 3 neck round bottom flask, ensuring that the temperature of the mixture remained below 15 °C. Dichloromethane (30 ml) was then added to the mixed acid. The magnetically stirred mixture was then cooled to 5 °C. Diethylene glycol (2.12 g) was slowly added (during 15 min) to the acid/dichloromethane mixture under vigorous stirring. Care was taken to ensure the temperature remained below 12 °C. After the addition was completed, the suspension was stirred for a further 30 min. It was then transferred to a separating funnel and the lower acid layer run off and discarded. The organic layer was then washed sequentially as follows: water (1 × 10 ml), 10% aqueous Na_2_CO_3_ (2 × 8 ml), 10% aqueous urea (1 × 10 ml), water (3 × 8 ml) and saturated brine (1 × 8 ml). The washed organic phase was dried over MgSO_4_, filtered and the solvent removed by rotary evaporation to leave the product, diethylene glycol dinitrate as a clear mobile oil. The purity of DEGDN was confirmed as 99.9% using ^1^H NMR spectroscopy.

### GO-doped NC/DEGDN films preparation

The GO-doped NC-DEGDN nanocomposite film was prepared in two stages. A 60/40 mixing ratio of NC : DEGDN in this work was chosen.

Stage 1: dissolution of a known quantity of NC (3 g) in butanone (100 ml). The mixture was left overnight to ensure swelling of NC in the solvent was completed. The dissolved NC was then stirred for 2 h to ensure optimum solvation.

The required quantities of GO (0.1–1.0% w/w of NC–DEGDN mixture) was weighed in a sealed vial containing butanone (5 ml) and subjected to ultrasonic agitation for 10 min. The suspended GO was then transferred to the NC solution followed by vigorous stirring overnight. The NC and GO suspension was then transferred to an ultrasonic bath where it was agitated for 10 min to ensure thorough exfoliation and even distribution of GO in NC solution. The DEGDN (2 g) was added slowly to the suspension followed by vigorously stirring for 12 h.

Stage 2: on to a spirit-levelled glass slide, known amounts of NC/DEGDN/GO solution was spread (2 ml per slide) using a micropipette. A series of films was prepared with different concentrations of GO. To control the rate of evaporation the glass slides were covered with Petri dishes with two small diameter holes on the top and left in a fume hood for 48 h. The films were protected from sunlight with aluminium foil. Once the majority of the solvent was evaporated the films were dried in a vacuum oven (50 °C) until a constant weight was achieved. The dried films were stored in a desiccator and used for testing.

### Characterization techniques

Atomic force micrographs (AFM) were acquired using a Digital Instruments Dimension™ 3000 SPM with a NanoScope IIIa controller (Veeco instruments, Santa Barbara, California, USA) and a NanoScope IIIa software (version 4.42r4). Tapping mode was employed across the surface of the samples at a frequency of 1 Hz.

The infrared (IR) spectra were recorded in the mid-infrared region (4000–550 cm^−1^) on a Bruker ALPHA in diamond attenuated total reflection (ATR) using Opus software. The resolution was 2 cm^−1^ at 128 scans.

Raman spectroscopy was performed on an iHR 320 model fully automated spectrometer with a wavelength position resolution of 0.2 cm^−1^. The excitation source was an argon ion laser (514 nm) within the wavenumber range of 0–4000 cm^−1^.

### Activation energy (*E*_a_) calculation

The thermal decomposition analysis was carried out using a Mettler Toledo DSC3+ device. We placed 10 mg of the material in a 40 μl aluminium DSC pan with a pierced lid. The DSC sample chamber was continuously purged with N_2_ gas at a flow rate of 50 ml min^−1^. The test temperature was selected to 50–250 °C. The variation of the heat flow at 1, 2, 5, and 10 °C min^−1^ respectively was recorded as a function of temperature and time.

The activation energies (*E*_a_) for the thermolytic decomposition of O–NO_2_ bonds of NC/DEGDN and GO/NC/DEGDN were calculated by applying Kissinger and Ozawa methods.^6,19^ Decomposition kinetics were obtained using two different models, which presented as follows:^1^

Kissinger method:1
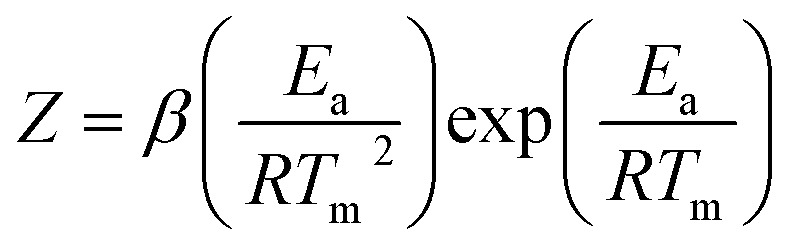


Ozawa method:2
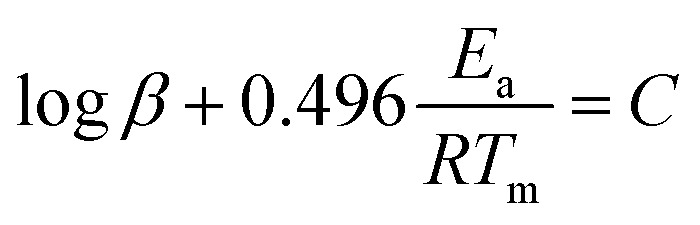
where *β* is the heating rate, *E*_a_ is the activation energy, *R* is the molar gas constant, *T*_m_ is the decomposition peak temperature of the DSC curve. DSC data were fitted in eqn (1) and (2), to calculate the *E*_a_ value from linear plots.

## Results and discussion

**Fig. 1:**
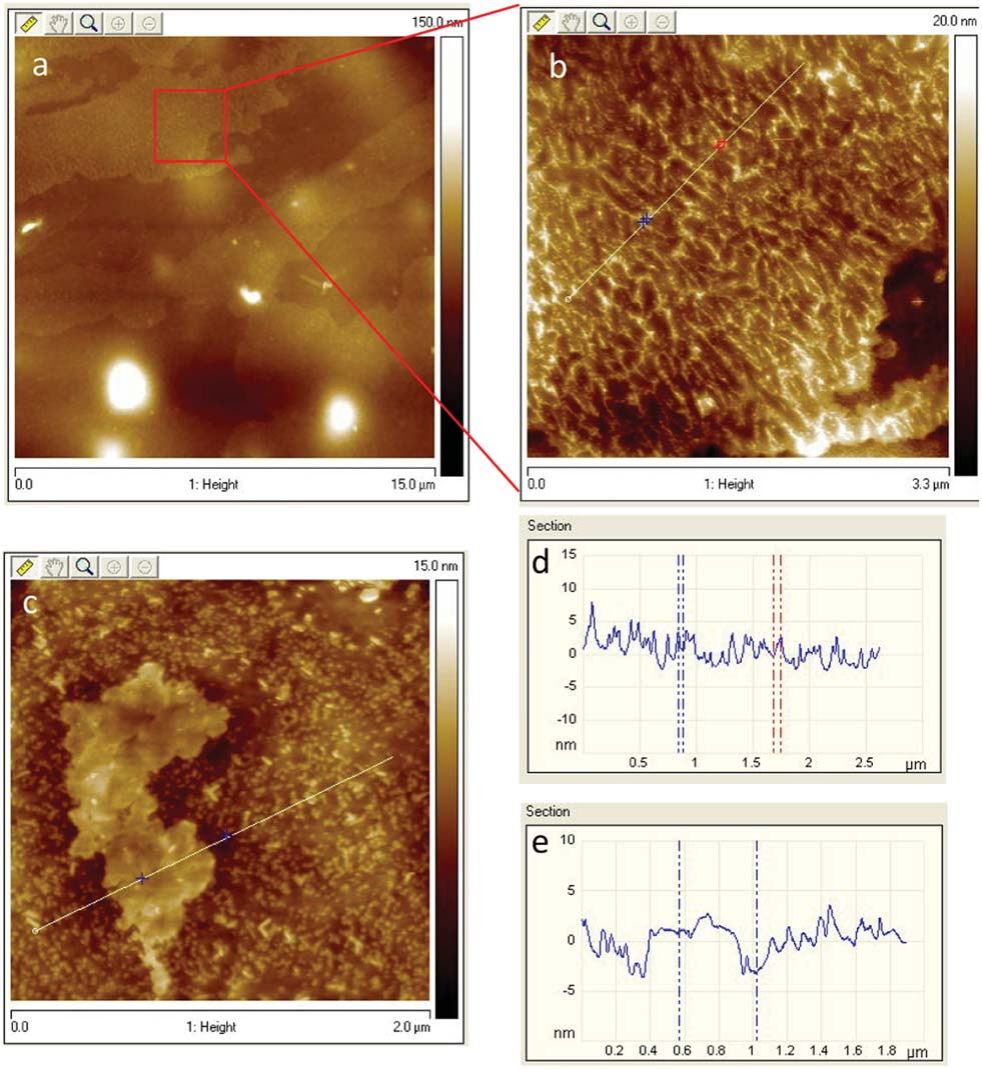
(a) AFM images of NC/DEGDN with 0.5% GO. In (b) zoomed image of 0.5% GO composition, (c) NC/DEGDN with 0.75% GO, (d) surface profile of image (a) and (e) is the surface profile of image (c).

To investigate the degree of exfoliation and dispersity of GO materials in NC/DEGDN, AFM images of the NC/DEGDN/GO were captured. The results are shown in the Fig. 1. The AFM images show that the GO was dispersed well in the NC/DEGDN. The presence of irregularly shaped sheets of uniform thickness and lateral dimensions ranging from a few hundred nanometres to a few micrometres can be seen in Fig. 1a–c. The surface topology confirms good exfoliation of 0.5% w/w GO sample into individual sheets in NC/DEGDN composite (Fig. 1a) suggesting DEGDN and NC are good host matrices for dispersion of GO. A necessary, but not sufficient, condition was thought to be the solvent polarity. This is reasonable because the GO sheets are heavily decorated with polar oxygen-containing functionalities such as hydroxyl groups, carbonyl groups, epoxy groups and carboxyl groups, which promote the host–guest interactions. Paredes *et al.* reported similar exfoliation results while GO was dispersed in DMF, THF, NMP and ethylene glycol.^24^

AFM image analysis also suggests NC/DEGDN matrix with 0.5% w/w GO shows an average of two layers of GO, given the fact that the thickness of a single-layer graphene is between 1.0–1.4 nm.^24^ However, as illustrated in the Fig. 1d and e for 0.75% w/w sample, an average of 3–4 layers of GO sheets are present. A thickness of 3–4 layers is attributed to the presence of oxygen-containing functional groups attached on both sides of the graphene sheet which then agglomerate through inter-sheet adhesion at higher than 0.5% of GO.^24^ Agglomeration of GO sheets is favoured at higher percentages.

The AFM images show the presence of wrinkles as seen in Fig. 1b. Minor wrinkles were frequently observed for the larger sheets.^24,25^ The interaction between oppositely charged GO and nitrate esters causes strong interlocking promoting wrinkles formation. Reports suggest that the tension between polar host molecules and GO strongly favours wrinkles formation.^26,27^

**Fig. 2:**
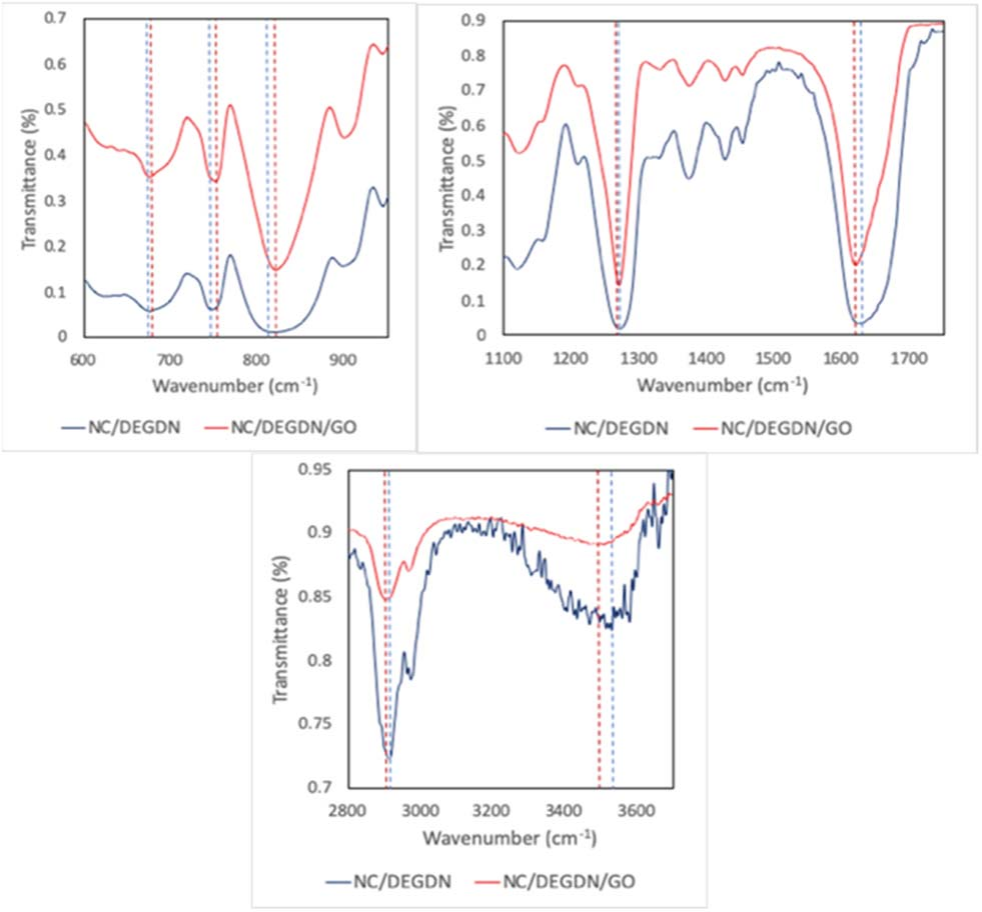
FTIR spectra of NC/DEGDN and NC/DEGDN/GO.

To confirm the multilayer GO structure and GO interaction with the host matrices, further studies were carried out on the NC/DEGDN/GO nanocomposites using FTIR and Raman spectroscopy. Fig. 2 shows a typical FTIR trace of NC/DEGDN and NC/DEGDN/GO samples. The FTIR band at 1620 cm^−1^ is assigned to asymmetric stretching vibration of N–O bond in –NO_2_, the band at 1270 cm^−1^ corresponds to symmetric vibration of –NO_2_ and the band at 814 cm^−1^ is assigned to O–NO_2_ stretching. The peaks at 1620 cm^−1^ and 1270 cm^−1^ remains unchanged in NC/DEGDN upon addition of GO, however peak at 814 red shifted to 808 cm^−1^ suggesting interaction of nitrate ester with the GO and that GO contributed electron to the electron deficient O–NO_2_ group making O–N bond more stable.^28,29^

The broad peak at 3520 cm^−1^ corresponding to the OH of NC in NC/DEGDN sample weakened and shifted to 3490 cm^−1^ for NC/DEGDN/GO sample spectra confirming hydrogen bonding between GO and OH groups in NC.^28,30^

The characteristic signals at 744 and 673 cm^−1^ are assigned to asymmetric deformation and symmetric deformation respectively of O–NO_2_ in the NC/DEGDN sample. These peaks blue shifted to 752 cm^−1^ and 683 cm^−1^ respectively suggesting interaction between NO_2_ and GO.^31^ The broad peak in 1000–1200 cm^−1^ region corresponds to the overlapped peaks for C–O–C bonds stretching vibration from the GO, NC and DEGDN.

Raman spectroscopy analysis was performed to characterize the structure of carbon in GO and NC/DEGDN/GO nanomaterials and the interaction of GO with nitrate esters.

**Fig. 3:**
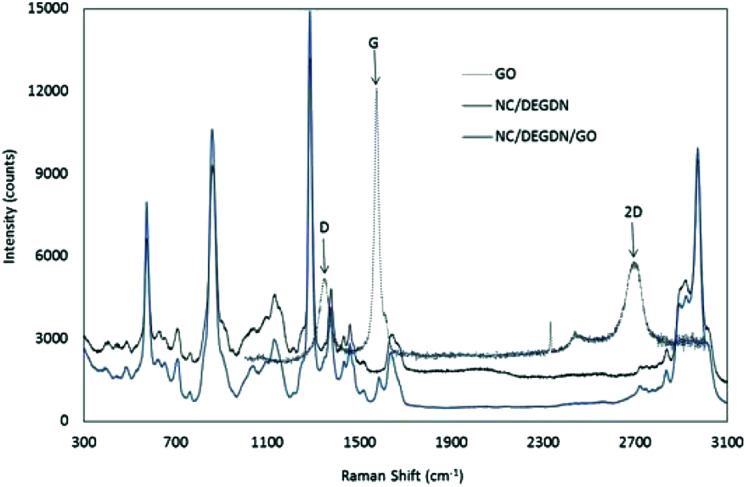
Raman spectra of NC, GO, NC/DEGDN and NC/DEGDN/GO.

Fig. 3 shows an overlaid Raman spectra of GO, NC/DEGDN and NC/DEGDN/GO composite. The peaks at 2970, 2916, 1640, 1375, 1283, and 814 cm^−1^ correspond to nitrocellulose and DEGDN.^32^ The details of the Raman peaks assignment are listed in Table 1.

**Table 1:** Raman spectra assignment of NC/DEGDN, GO and NC/DEGDN/GO

NC/DEGDN	Nanocomposite	GO	Characteristics
1640	1620	—	NO_2_ asymmetric
	1583	1572	G bands
1376	1373 (sp^3^ of NC ring mode)	1344	D bands
1283	1283		NO_2_ symmetric
859	859		O–N vibration
—	2718	2693	2D bands
2916	2890	—	CH
2970	2970	—	CH_2_

From the Raman spectra data in Table 1 it can be seen that the GO gives 3 signature peaks known as G, D and 2D bands.^33–35^ In GO compound, the Stokes phonon energy shift is caused by laser excitation which causes GO to have the main peaks namely G band and D bands.

The peak at 1572 cm^−1^ namely a primary in plane vibrational mode corresponds to G band, a second order overtone at 1344 cm^−1^ in a different plane vibration. The peak at 2693 cm^−1^ corresponds to 2D band associated with second order plane vibration.^25–27^

Upon addition of GO in NC/DEGDN composition G band at 1572 cm^−1^ blue shifted to 1583 cm^−1^. Similarly D band and 2D bands also blue shifted from 1344 cm^−1^ and 2693 cm^−1^ respectively to 1373 cm^−1^ and 2718 cm^−1^ suggesting reduction of electron from GO structure which was accepted by the electron deficient O–NO_2_ groups (donor–accepter interaction) of NC and DEGDN. Other interactions such as π–π interaction of π-bond of GO and –NO_2_ and electrostatic adhesion of GO with the negatively charged oxygen of NO_2_ groups have also been reported.^36^

Furthermore the Raman spectrum of NC/DEGDN/GO shows peak broadening and significant decrease in the intensity of G and D bands suggesting successful dispersion of GO in the NC/DEGDN matrix and interaction between them.^37^

The spectra also confirm the hydrogen bonds between NC/DEGDN and GO. The D and G bands arise due to skeletal vibrations of GO at the sp^3^ and sp^2^ rich domain respectively. The ratio of D/G indicates the formation of defects in the GO sheets which are considered to be π–π interaction between GO and nitrate ester groups (O–NO_2_). Such an interaction with host matrices generates wrinkles on the GO surface which in this case is evident from AFM images in Fig. 1.^26,27^

We compared the ratio of D/G band from NC/DEGDN with various GO concentration (0.25, 0.5, 0.75, 1.0%) and results shown in Table 2.

**Table 2:** D/G bands ratio of NC/DEGDN with various GO%^a^

GO (%)	Raman shift (cm^−1^)		D/G ratio
	D bands	G bands	
0.25	1375	1633	1.63
0.50	1373	1583	2.57
0.75	1367	1583	1.23
1.00	1365	1584	1.02

a Composite refers to NC/DEGDN/GO.

As can be seen in Table 2 the ratio of D and G bands increased up to a threshold amount of 0.5% GO content suggesting increased dis-orderness of sp^3^ character in the GO which related to the formation of H-bonding between GO and NC/DEGDN. Note that 12.6% nitrogen containing NC was not completely nitrated leaving some OH groups un-nitrated which involved in hydrogen bonding with GO. However, the D/G ratio decreased above 0.5% GO loading suggesting lowering of sp^3^ defects. This can best be interpreted as the GO concentration increased inter-layer adhesion of GO dominates over the adhesion of GO and NC/DEGDN matrix. This is evidenced in the AFM image (Fig. 1) where a 0.75% GO containing sample shows multilayer GO sheets.

**Fig. 4:**
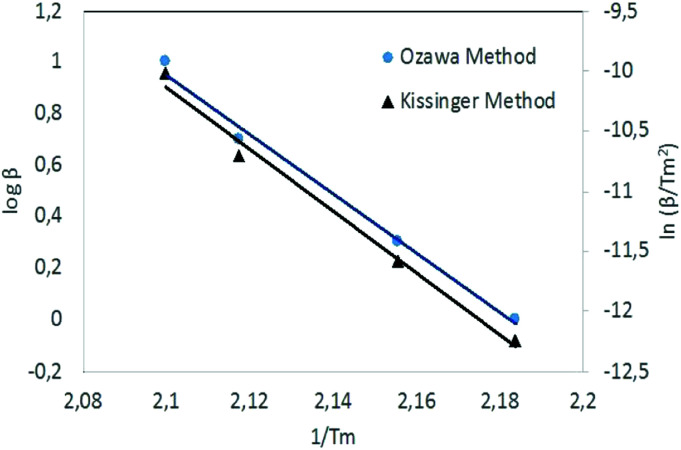
Kissinger's plot for determination of the activation energy *E*_a_ from a set of DSC scans on nanocomposite with 0.5% GO.

Kissinger and Ozawa plots use a set of DSC scans with different heating rates (1, 2, 5 and 10 °C min^−1^ in this case). The plots according to eqn (1) and (2) are straight lines for NC/DEGDN and NC/DEGDN/GO with various GO ratio 0.25–1.0%, suggesting that the decomposition follows thermolytic first order reaction mechanism.^26^Fig. 4 shows a typical Kissinger and Ozawa plots for the nanocomposite with 0.5% GO.

**Fig. 5:**
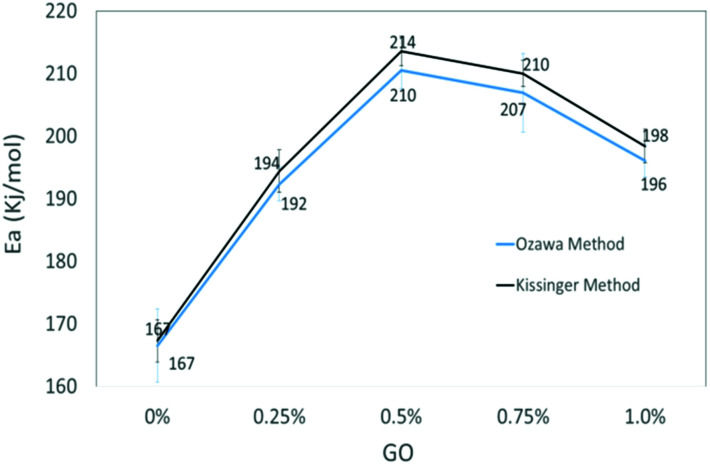
Comparison activation energy (*E*_a_) of NC/DEGDN with various % GO.

The slopes of the two straight lines were used to calculate the activation energy for the decomposition of O–NO_2_ groups. Activation energy as a function of GO loading in NC/DEGDN matrices are shown in Fig. 5.

The activation energy of thermolytic decomposition of was found to about 167 ± 3.3 kJ mol^−1^ which is in-line with the literature value.^38^ Another trend in Fig. 5 shows that the activation energy of nitrate ester decomposition increased to a maximum value of 214 ± 2.4 kJ mol^−1^ with 0.5% GO content and then started decreasing above the threshold value.

The activation energy of decomposition of nitrate ester bonds in NC/DEGDN increases with GO loading, with a maximum increase of 28% with 0.5% GO. Subsequent addition of GO in NC/DEGDN showed a decrease of the activation energy which is attributed to reduced interaction of GO with the matrix due to dominant inter-layer interaction and agglomeration of GO sheets themselves. Linearity of the plots of all the GO doped and pure samples indicates that the reaction order of NC/DEGDN and GO/NC/DEGDN was similar. The symmetry of the decomposition peaks of the DSC curves of the films did not change as a function of the percentage GO indicating that the addition of GO did not change the decomposition mechanism of NC/DEGDN.

**Fig. 6:**
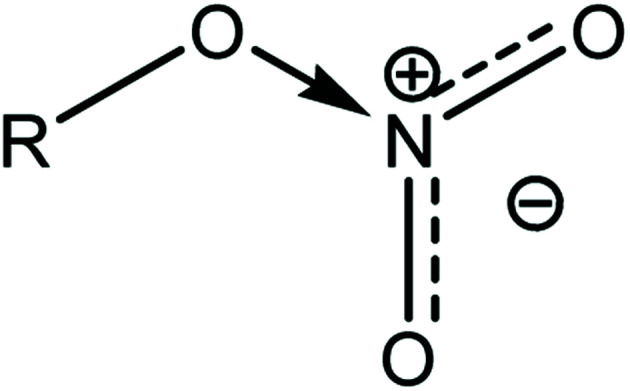
Delocalised nitrate ester group.

An increase in activation energy suggests that the nitrate ester bonds (O–NO_2_) in NC/DEGDN are stabilized by the delocalisation of GO electrons to the electron deficient nitrate ester groups, which has been explained by the blue shift of D and G bands and an increase of D/G band ratio of NC/DEGDN/GO up to 0.5% GO loading in Table 2. The O–NO_2_ group in NC and DEGDN have resonance structure^39^ as shown in Fig. 6.

**Fig. 7:**
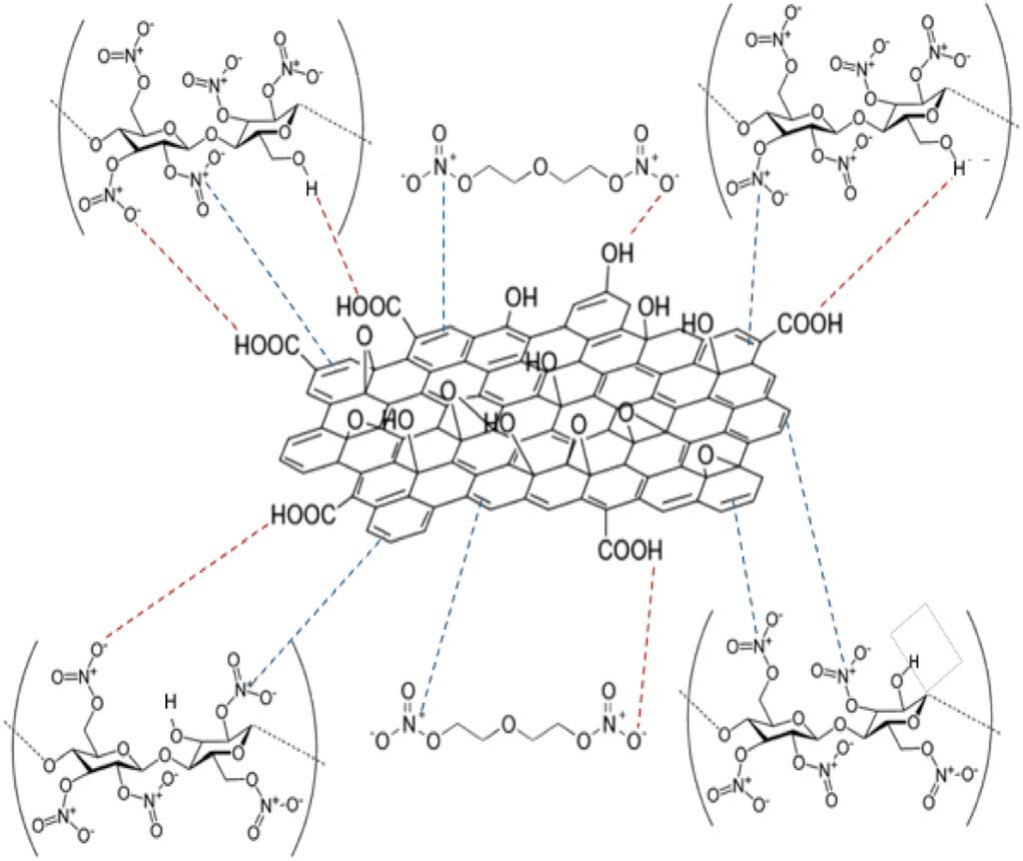
Schematic illustration of GO and NC/DEGDN complex.

The delocalised electron in N–O bonds of nitro groups makes the nitrogen atoms positively charged. The positively charged nitrogen atom attracts electrons from the O–N bond making it less stable, therefore breaks at a lower energy (activation energy). On the other hand GO is enriched with electrons and acts as an electron donor. The donation of electrons to the electron deficient O–NO_2_ makes the O–N bond more stable, thus requiring larger energy to break the O–N bond. Further stabilisation can be caused by a number of reasons such as interfacial adhesion of NC/DEGDN with electron rich GO, hydrogen bonding and electrostatic interlocking of matrix. All these interactions have already been reported elsewhere and, we discussed in the AFM, FTIR and Raman spectra sections in this paper. The decrease of activation energy above 0.5% GO loading is thought to be due to agglomeration of GO by interlayer adhesion of GO sheets; this adhesion is amplified by an increasing amount of GO in the matrix since at higher loading, GO sheets are too close to each other, facilitating interlayer agglomeration and reducing GO effect on host matrix. This has been claimed by observing multilayers of GO in the AFM image of the sample with 0.75% GO, suggesting lowering of interaction between GO and nitrate esters. It is speculated that the complex structure of NC/DEGDN/GO is somewhere like the one shown Fig. 7.

## Conclusions

A new NC/DEGDN/GO energetic nanocomposite has been successfully prepared through a GO dispersion in a nitrate ester mixture of NC and DEGDN formulation. AFM spectra analysis suggested a good dispersion of GO in the matrix with the number of layers ranging from 1 to 4. 0.5% GO content sample showing 1–2 layers and above 0.5% showing multilayer GO sheets. The presence of wrinkles in the AFM image confirmed sp^3^ defects due to the presence of electron holes by the functional groups in GO and interaction between GO and host matrix. The FTIR and Raman spectroscopy confirmed the donor-accepter, electrostatic, possibly π–π and hydrogen bonding interactions between GO and NC/DEGDN matrix which ultimately reduced the thermolytic decomposition of nitrate ester bond as confirmed by the increase of activation energy by about 28% with 0.5% GO. Further addition of GO in the matrix decreases activation energy due to reduced interaction between GO and the NC/DEGDN matrix. The importance of this research finding is that this will open a new direction to the application of graphene and its derivatives in the nitrate ester-based materials used in lacquer, cosmetics and munitions for improving stability, safety, performance and shelf life.

## Conflicts of interest

There are no conflicts to declare.

## Supplementary Material
